# Non-IDH1-R132H IDH1/2 mutations are associated with increased DNA methylation and improved survival in astrocytomas, compared to IDH1-R132H mutations

**DOI:** 10.1007/s00401-021-02291-6

**Published:** 2021-03-19

**Authors:** C. Mircea S. Tesileanu, Wies R. Vallentgoed, Marc Sanson, Walter Taal, Paul M. Clement, Wolfgang Wick, Alba Ariela Brandes, Jean Francais Baurain, Olivier L. Chinot, Helen Wheeler, Sanjeev Gill, Matthew Griffin, Leland Rogers, Roberta Rudà, Michael Weller, Catherine McBain, Jaap Reijneveld, Roelien H. Enting, Francesca Caparrotti, Thierry Lesimple, Susan Clenton, Anja Gijtenbeek, Elizabeth Lim, Filip de Vos, Paul J. Mulholland, Martin J. B. Taphoorn, Iris de Heer, Youri Hoogstrate, Maurice de Wit, Lorenzo Boggiani, Sanne Venneker, Jan Oosting, Judith V. M. G. Bovée, Sara Erridge, Michael A. Vogelbaum, Anna K. Nowak, Warren P. Mason, Johan M. Kros, Pieter Wesseling, Ken Aldape, Robert B. Jenkins, Hendrikus J. Dubbink, Brigitta Baumert, Vassilis Golfinopoulos, Thierry Gorlia, Martin van den Bent, Pim J. French

**Affiliations:** 1 Department of Neurology, Brain Tumor Center at Erasmus MC Cancer Institute Rotterdam, PO Box 2040, 3000 CA Rotterdam, The Netherlands; 2 Sorbonne Universités UPMC University of Paris 06, Inserm, CNRS, APHP, Institut du Cerveau et de la Moelle (ICM)-Hôpital Pitié-Salpêtrière, Boulevard de l’hôpital, 75013 Paris, France; 3 Department of Oncology, KU Leuven, Leuven, Belgium; 4 Department of General Medical Oncology, UZ Leuven, Leuven, Belgium; 5 Neurologische Klinik und Nationales Zentrum für Tumorerkrankungen Universitätsklinik, Heidelberg, Germany; 6 Medical Oncology Department, AUSL-IRCCS Scienze Neurologiche, Bologna, Italy; 7 Medical Oncology Department, King Albert II Cancer Institute, Cliniques Universitaires Saint-Luc, Université Catholique de Louvain, Bruxelles, Belgium; 8 Neuro-Oncology Division, Aix-Marseille University, AP-HM, Marseille, France; 9 Northern Sydney Cancer Centre, St Leonards, NSW 2065 Australia; 10 Department Medical Oncology, Alfred Hospital, Melbourne, Australia; 11 Department of Clinical Oncology, Nottingham University Hospitals NHS Trust, Nottingham, UK; 12 Department of Radiation Oncology, Barrow Neurological Institute, Phoenix, AZ USA; 13 Department of Neuro-Oncology, City of Health and Science Hospital and University of Turin, Turin, Italy; 14 Department of Neurology and Brain Tumor Center, University Hospital and University of Zurich, Zurich, Switzerland; 15 Department of Clinical Oncology, The Christie NHS FT, Manchester, UK; 16 Brain Tumor Center Amsterdam and Department of Neurology, Amsterdam University Medical Center, Amsterdam, The Netherlands; 17 Department of Neurology, UMCG, University of Groningen, Groningen, The Netherlands; 18 Department of Radiation Oncology, University Hospital of Geneva, Geneva, Switzerland; 19 Department of Clinical Oncology, Comprehensive Cancer Center Eugène Marquis, Rennes, France; 20 Weston Park Hospital, Sheffield, UK; 21 Department of Neurology, Radboud University Medical Centre, Nijmegen, The Netherlands; 22 Department of Clinical Oncology, Plymouth Hospitals NHS Trust, Plymouth, UK; 23 Department of Medical Oncology, UMC Utrecht Cancer Center, Utrecht, The Netherlands; 24 University College Hospital, London, UK; 25 MC Haaglanden, Den Haag, The Netherlands; 26 Department of Pathology, Leiden University Medical Center, Leiden, The Netherlands; 27 Edinburgh Centre for Neuro-Oncology, Western General Hospital, University of Edinburgh, Edinburg, UK; 28 Department of NeuroOncology, Moffitt Cancer Center, Tampa, FL USA; 29 School of Medicine and Pharmacology, University of Western Australia, 35 Stirling, Highway Crawley, WA 6009 Australia; 30 CoOperative Group for NeuroOncology, University of Sydney, Camperdown, NSW Australia; 31 Department of Medical Oncology, Sir Charles Gairdner Hospital, Hospital Avenue, Nedlands, WA 6009 Australia; 32 Princess Margaret Cancer Centre, University of Toronto, Toronto, Canada; 33 Department of Pathology, Erasmus University Medical Center, Rotterdam, The Netherlands; 34 Department of Pathology, VU University Medical Center, Amsterdam, The Netherlands; 35 Department of Laboratory Medicine and Pathology, Mayo Clinic, Rochester, MN USA; 36 Department of Radiation-Oncology (MAASTRO), Maastricht University Medical Center (MUMC) and GROW (School for Oncology), Maastricht, The Netherlands; 37 Institute of Radiation-Onology, Chur, Switzerland; 38 EORCT HQ, Brussels, Belgium

**Keywords:** Astrocytoma, Genome-wide DNA methylation, Gene expression, IDH1, IDH2

## Abstract

Somatic mutations in the isocitrate dehydrogenase genes *IDH1* and *IDH2* occur at high frequency in several tumour types. Even though these mutations are confined to distinct hotspots, we show that gliomas are the only tumour type with an exceptionally high percentage of IDH1^R132H^ mutations. Patients harbouring IDH1^R132H^ mutated tumours have lower levels of genome-wide DNA-methylation, and an associated increased gene expression, compared to tumours with other IDH1/2 mutations (“non-R132H IDH1/2 mutations”). This reduced methylation is seen in multiple tumour types and thus appears independent of the site of origin. For 1p/19q non-codeleted glioma (astrocytoma) patients, we show that this difference is clinically relevant: in samples of the randomised phase III CATNON trial, patients harbouring tumours with IDH mutations other than IDH1^R132H^ have a better outcome (hazard ratio 0.41, 95% CI [0.24, 0.71], *p* = 0.0013). Such non-R132H IDH1/2-mutated tumours also had a significantly lower proportion of tumours assigned to prognostically poor DNA-methylation classes (*p* < 0.001). IDH mutation-type was independent in a multivariable model containing known clinical and molecular prognostic factors. To confirm these observations, we validated the prognostic effect of IDH mutation type on a large independent dataset. The observation that non-R132H IDH1/2-mutated astrocytomas have a more favourable prognosis than their IDH1^R132H^ mutated counterpart indicates that not all IDH-mutations are identical. This difference is clinically relevant and should be taken into account for patient prognostication.

**Supplementary Information:**

The online version contains supplementary material available at 10.1007/s00401-021-02291-6.

## Introduction

Somatic mutations in the isocitrate dehydrogenase genes *IDH1* and *IDH2* occur at high frequency in various tumour types including gliomas (primary malignant central nervous system tumours), intrahepatic cholangiocarcinomas (bile duct tumours), enchondromas and chondrosarcomas (bone tumours), sinonasal undifferentiated carcinomas and leukemias [12, 33]. More sporadic but similar mutations have been found in a wide variety of other tumour types including melanoma, and prostate and pancreatic cancer [54]. *IDH1/2* mutations are causal for the disease and tumours often remain dependent on the mutation for growth [22, 42]. The importance of the mutation is confirmed by the activity of IDH-inhibitors: inhibiting the mutant activity of either *IDH1* or *IDH2* shows anti-tumour activity in relapsed/refractory *IDH1/*2 mutated acute myeloid leukemia [14, 45] and cholangiocarcinoma patients [1]. The objective response rates in these trials are in the order of 40%, though patients eventually relapse. In gliomas, however, mutant *IDH1/2* inhibitors have thus far not shown a survival benefit, but further studies on early-stage tumours are ongoing [32].

The *IDH1/2* mutations are confined to defined gene hotspots and affect either arginine 132 (R132) in *IDH1* or arginines R172 or R140 in *IDH2*. Although *IDH1/2* mutations are confined to these three hotspots, several reports have shown that the IDH-mutation spectrum differs per tumour type [12, 15, 20, 37]. The hotspot mutations all change the activity of the wild-type (wt) protein from an enzyme that produces alpha-ketoglutarate (aKG) to an enzyme that produces D-2 hydroxyglutarate (D-2HG) [12, 27] which ultimately keeps cells in an undifferentiated state [19, 30], but individual IDH1/2 mutations differ in their ability to produce D-2HG [5, 40]. IDH1^R132H^, the *IDH1/2* mutation with relatively low D-2HG production capacity, is the most common mutation in gliomas; other mutations such as IDH1^R132C^ have tenfold lower *K*_M_ and have higher enzymatic efficiency [5, 40]. This difference may have biological implications as not all aKG-dependent enzymes are equally well inhibited by D-2HG [11, 53]. For example, tet methylcytosine dioxygenase 2 (TET2) enzymes that mediate the first step in DNA-demethylation, requires relatively high D-2HG levels for inhibition [31, 53].

Here, we have used data from six large and independent DNA methylation datasets (the randomised phase III CATNON clinical trial on anaplastic 1p/19q non-codeleted gliomas [49], the TCGA-LGG cohort [8], samples included in the TAVAREC randomised phase 2 clinical trial on astrocytomas [51], a large cohort of acute myeloid leukemias (AML) [48] and a cohort of chondrosarcomas [52]) derived from four different tumour types, to examine the molecular effects of different types of *IDH1/2* mutations. We report that tumours harbouring IDH1^R132H^ mutations, regardless of tumour type, have lower genome-wide DNA methylation levels compared to those harbouring other *IDH1/2* hotspot mutations (‘non-IDH1-R132H *IDH1/2*-mutated tumours’). For astrocytoma patients, we show this difference has clinical relevance as patients harbouring such non-IDH1R132H IDH1/2-mutated tumours have improved survival compared to those harbouring IDH1^R132H^ mutations. Our data support the notion that increased genome-wide DNA methylation levels are associated with improved outcome in this tumour type and indicate that the type of *IDH1/2* mutation should be taken into account for prognostication of astrocytoma patients.

## Materials and methods

### Datasets

The COSMIC database (Assessed 27 December 2019) was screened for hotspot *IDH1* (R132) and *IDH2* (R172 and R140) mutations. Mutations were stratified by tumour type; tumours with a low prevalence of mutations were concatenated (site of origin of ‘other tumours’: prostate *n* = 11, pancreas *n* = 6, skin *n* = 32, large intestine *n* = 1, soft tissue *n* = 22, endometrium *n* = 1, breast *n* = 9, urinary tract *n* = 2, liver *n* = 7, stomach *n* = 1, upper aerodigestive tract *n* = 35, salivary gland *n* = 1, thyroid *n* = 1). CATNON clinical data [49] and *IDH1/2* mutation and DNA methylation data (Tesileanu, submitted) were reported previously. TCGA glioma data (DNA methylation and RNA-seq) [8], MSK-IMPACT data [9] and AML data [48] were downloaded from the TCGA data portal. Clinical data and mutation status for the chondrosarcoma data were reported previously [52]. Clinical data from the TAVAREC trial were derived from ref [51], and supplemented with DNA methylation data of 89 tumours. Most (80%) TAVAREC samples were derived from the initial tumour. Processing of CATNON and TAVAREC DNA methylation data was performed as described (Tesileanu, submitted). For the CATNON, TCGA-astrocytoma and TAVAREC datasets, we included only *IDH1/2* mutated samples from non 1p/19q-codeleted tumours. Although all CATNON and TAVAREC samples were initially diagnosed as astrocytomas, DNA methylation analysis found 1p/19q codeletion in 8 samples included in the CATNON trial and 3 samples in the TAVAREC trial (Tesileanu, submitted). To ensure a molecularly homogenous sample cohort, all 1p/19q codeleted samples were removed prior to any analysis presented. For *IDH1/2* mutated MSK-IMPACT samples, the distinction between astrocytic and oligodendrocytic tumours was made by absence or presence of telomerase reverse transcriptase (TERT) promoter mutations [26, 46]. In the Chinese Glioma Genome Atlas [CGGA] [23], the exact *IDH1/2*-mutation was not noted and therefore limited for the scope of this analysis. We used only the 1p/19q codeleted tumours in this dataset with *IDH2* mutations being designated as “non-IDH1R132H IDH1/2-mutations” and all *IDH1* mutations as “R132H”. In oligodendrogliomas, IDH1 mutations virtually always result in R132H [20]. RNA-seq data (raw read counts) were normalized and processed using DEseq2.

### Statistical analysis

Survival curves were created using the Kaplan–Meier method. The log-rank test was used to determine survival differences. A Wilcoxon rank test on beta values (i.e. the intensity of the methylated probe/sum of methylated and unmethylated probe intensity) was used to identify differentially methylated probes in CATNON and TCGA-astrocytoma datasets. To increase power in the smaller sized datasets, we performed an *F* test on *M* values (i.e. the log2 ratio of the methylated/unmethylated probe intensities) to identify differentially methylated CpGs using the dmpFinder function in the Minfi Bioconductor package [4]. To further increase statistical power in the chondrosarcoma dataset (required as this dataset had few samples), we first made a selection of the most variable probes (i.e. those with a standard deviation > 2; ~ 5% of the total number of probes) followed by an *F* test on the *M* values. In all differential methylation analysis, p-values were corrected for false discovery rate (adjusted p-value).

Differences in mutation frequencies were determined using a chi-squared test. Pathway analysis was performed using Ingenuity pathway analysis (Qiagen, Venlo, The Netherlands). An association model was made with the Cox proportional hazards method and included, next to *IDH1/*2 mutation type, factors that are known to be related to outcome from literature such as sex, treatment with temozolomide, age at randomization, WHO performance score, *O*^6^-methylguanine DNA methyltransferase (*MGMT*) promoter methylation status, use of corticosteroids at randomization, and DNA methylation profiling. All *p* values below 0.05 were considered significant. Statistical analysis was performed using R version 3.6.3 and packages minfi, stats, rms, survival.

## Results

### *The IDH1*^*R132H*^* mutation predominates in gliomas*

**Fig. 1:**
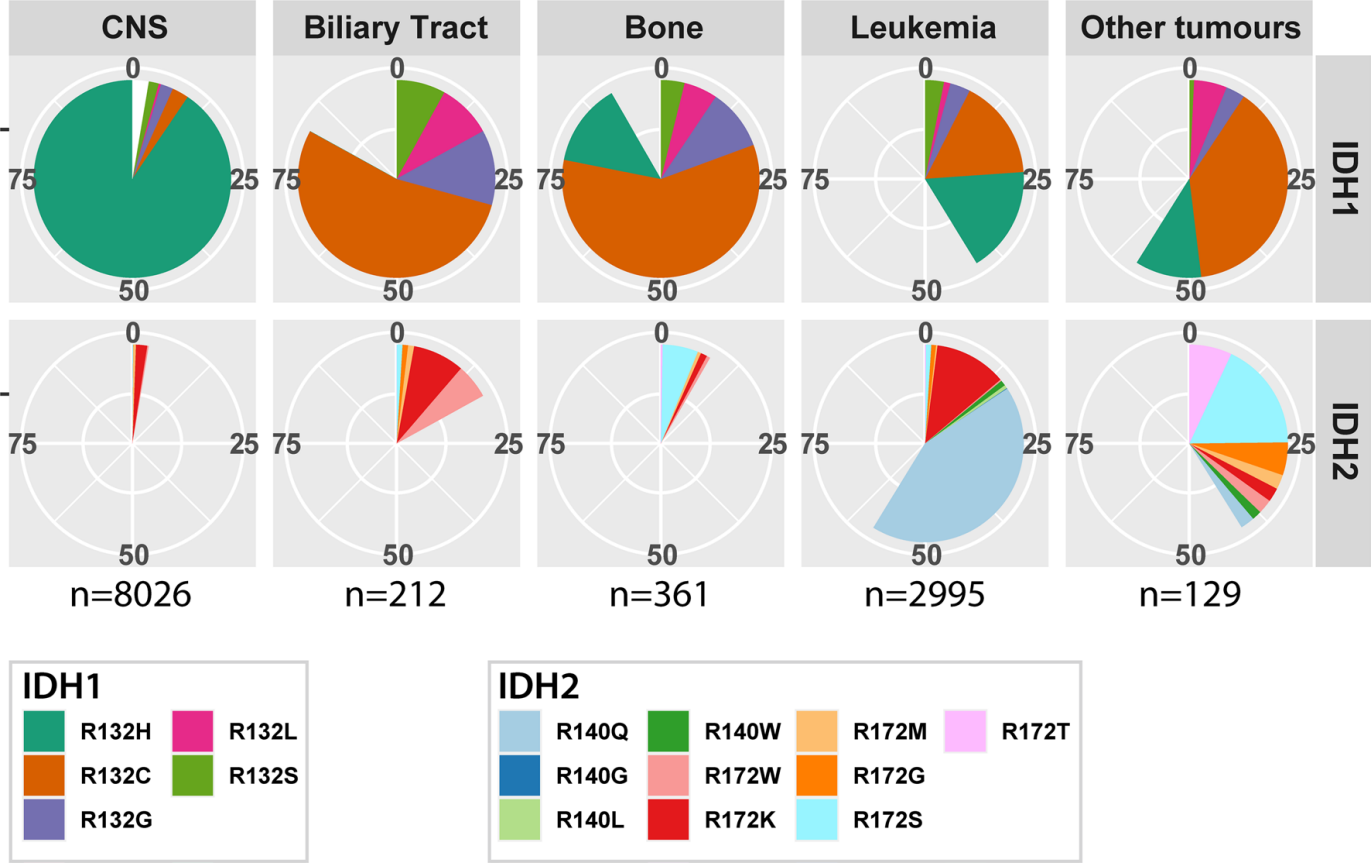
*IDH1* and *IDH2* hotspot mutation distribution separated by site of origin. IDH1^R132H^ mutations are the most predominant mutation in gliomas, IDH2 mutations are most common to haematopoietic tumours

We screened the catalogue of somatic mutations in the cancer (COSMIC) database [16], extracted *IDH1/2* hotspot mutation data (IDH1^R132^, IDH2^R172^ and IDH2^R140^) and stratified them by tumour organ site. As expected, tumours with a high frequency of *IDH1/2* mutations include the central nervous system (CNS), biliary tract, bone, haematopoietic and lymphoid tumours (leukemias). Interestingly, even if there are only three mutational hotspots, there are marked differences in the distribution of mutations between tumour sites (Fig. 1). For example, the IDH1^R132H^ mutation is by far the most predominant IDH1/2 mutation in CNS tumours (*n* = 7265/8026, 90.5%) whereas this mutation is present at much lower frequencies in bone (*n* = 49/361, 13.6%), leukemic (*n* = 519/2995, 17.3%) and other tumours (*n* = 14/129, 10.9%), and thus far has never been identified in biliary tract tumours (*n* = 212) (*p* < 0.001, chi-square test). In contrast, the mutation that results in IDH1^R132C^ is quite rare in gliomas (223/8026, 2.8%) but much more prevalent in all other tumour types: bone (*n* = 212/361, 67.1%), leukemic (*n* = 493/2995, 16.5%), biliary tract (*n* = 114/212, 53.8%) and other tumours (*n* = 14/129, 10.9%). There is also a major difference in the distribution of *IDH2* mutations which are very common in haematopoietic and lymphoid tumours but rare in all other tumour types. Mutations of the R140 in *IDH2* are virtually exclusive to haematopoietic and lymphoid tumours.

### *DNA methylation is lower in IDH1*^*R132H*^* mutant glioma*

**Fig. 2:**
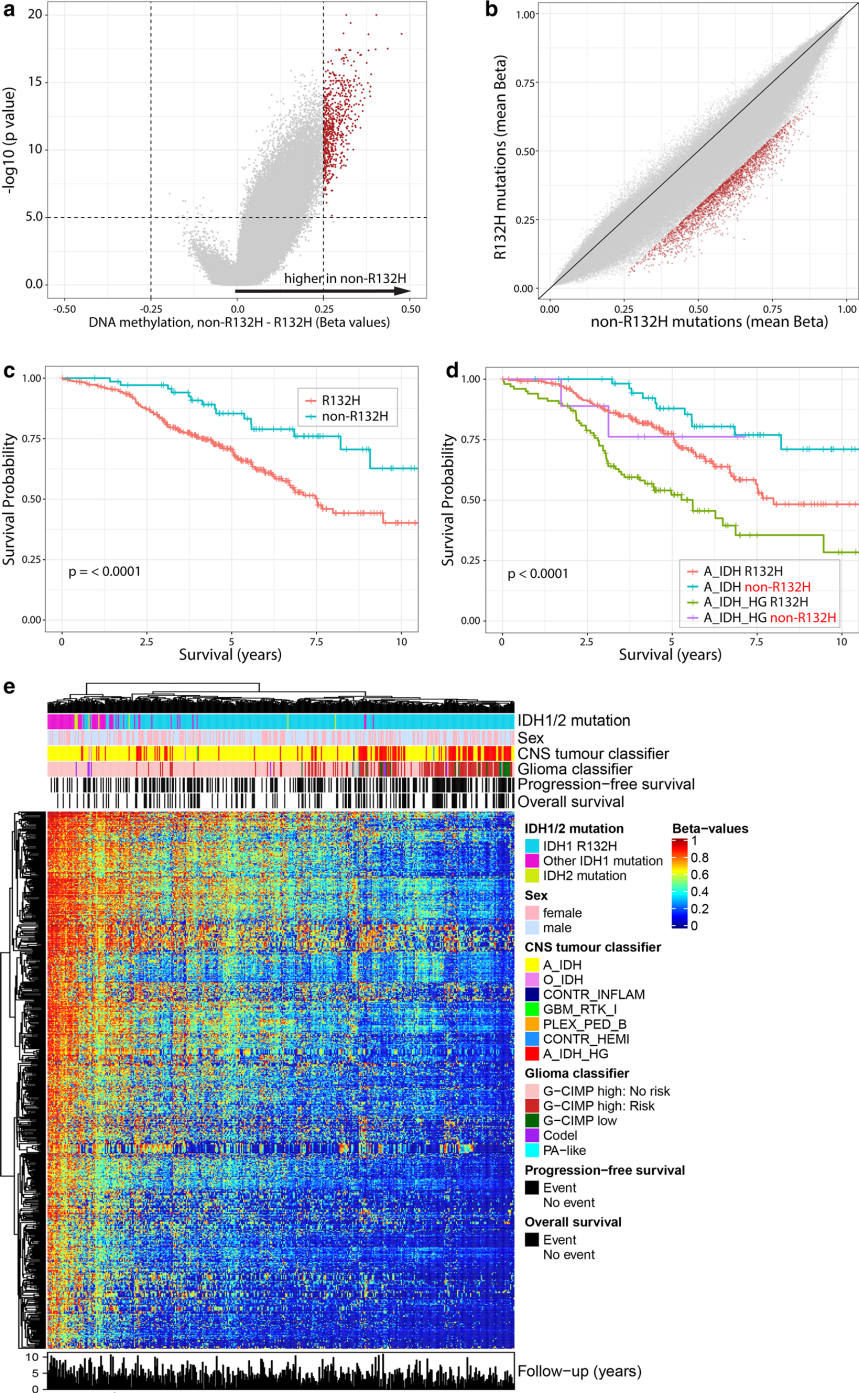
Non-R132H IDH1/2-mutations are associated with higher DNA methylation levels and improved survival of 1p19q non-codeleted astrocytoma patients included in the CATNON trial. Volcano plot (**a**) and XY plot (**b**) showing differences in methylation in non-R132H IDH1/2 vs. IDH1^R132H^ mutated tumours. **c** Patients harbouring non-R132H IDH1/2-mutated tumours have improved outcome, which is independent of methylation class (**d**). **e** Heatmap of the most differentially methylated probes (red dots in **a** and **b**), shows a gradient in methylation levels. Non-R132H IDH1/2-mutated tumours cluster at the far left (high methylation), where poor prognostic methylation subtypes (epigenetics subtypes) cluster at the opposite end

We used genome-wide DNA methylation data from CATNON trial samples and compared profiles of IDH1^R132H^ mutated tumours (*n* = 369) to those harbouring other “non-R132H” *IDH1* and *IDH2* hotspot mutations (*n* = 69). Our data shows that the overall level of DNA methylation was significantly lower in tumours harbouring IDH1^R132H^ mutations compared to tumours harbouring non-IDH1R132H IDH1/2-mutations. For example, there are 2461 probes showing a reduction in beta values > 0.2 in IDH1^R132H^ mutated tumours (at *p* < 0.01) but there are no probes showing an increase > 0.2. This is exemplified in the volcano plot where a strong skew towards increased DNA methylation in non-IDH1R132H IDH1/2- mutated samples is observed (Fig. 2a). Probes showing the largest increase in DNA methylation were those that were partially methylated in IDH1^R132H^ mutated tumours (i.e. probes with beta values between 0.25 and 0.75); there were few probes that became (partially) methylated from an unmethylated state (Fig. 2b).

Gliomas with higher levels of genome-wide DNA methylation generally are associated with longer survival in adults [8, 13, 28, 35]. Since non-R132H IDH1/2-mutated gliomas have increased DNA methylation levels, we compared the overall survival of patients with different IDH mutations. In patients included in the CATNON randomised phase III clinical trial, those harbouring tumours with non-R132H IDH1/2-mutations indeed had longer overall survival compared to patients harbouring IDH1^R132H^ mutated tumours (Fig. 2c). The hazard ratio for non-R132H IDH1/2-mutations compared to IDH1^R132H^ mutations was 0.41, 95% CI [0.24, 0.71], *p* = 0.0013.

DNA methylation profiling can also assign tumours to specific (prognostic) methylation subclasses. In line with the poorer survival, IDH1^R132H^ mutated tumours also had a significantly higher proportion assigned to the prognostically poorer subclass A_IDH_HG (“IDH-mutant, high-grade astrocytoma”, *n* = 100/366 vs. 9/71, *p* = 0.036, Chi-squared test) using the subclasses as defined by Capper et al. (“CNS-classifier”) [7]. They also have a higher proportion of G-CIMP low tumours (18/369 vs. 0/62) and G-CIMP-high tumours with risk to progression to G-CIMP low (111/335 vs. 2/62) in the classifier as defined by the TCGA and de Souza et al. (“glioma classifier”, *p* < 0.001, chi-squared test, Table 1) [8, 13].

**Table 1:** Multivariable model

	HR	95% CI		*p* value
IDH mutation type				
Non-R132H v. R132H	0.486	0.278	0.852	0.012
Sex				
Male v. female	1.465	1.033	2.076	0.032
Treatment				
RT → TMZ vs. RT	0.410	0.257	0.653	0.000
TMZ/RT vs. RT	0.802	0.520	1.237	0.319
TMZ/RT → TMZ vs. RT	0.385	0.231	0.639	0.000
Age				
40–60 vs. < 40 years	1.121	0.656	1.914	0.677
> 60 vs. < 40 years	3.824	1.812	8.069	0.000
Performance score				
1 vs. 0	1.404	0.991	1.990	0.056
2 vs. 0	2.282	0.704	7.401	0.169
MGMT promoter methylation				
UM vs. M	1.001	0.640	1.567	0.996
Corticosteroid use				
Yes vs. no	1.099	0.742	1.627	0.639
Methylation subtype				
A_IDH_HG vs. A_IDH	2.650	1.828	3.842	0.000
O_IDH vs. A_IDH	0.362	0.083	1.584	0.177
Other vs. A_IDH	10.763	3.410	33.970	0.000

A heatmap of the most differentially methylated CpGs of CATNON data (*n* = 677, selected on a beta value change > 0.25 and false discovery corrected *p* values < 10e−5) shows a gradient from high to low methylation levels. As expected, the non-R132H IDH1/2-mutated tumours cluster together at the high-methylation end of this spectrum. Interestingly, most of the tumours with less favourable molecular subtypes (A_IDH_HG, G-CIMP low, G-CIMP high with risk to progression) clustered together at the other, demethylated end (Fig. 2e). Although the clinical follow-up of CATNON patients is limited, the number of mortality events also tended to cluster at the demethylated end of the heatmap which suggests that there is a strong correlation between the level of methylation of these 677 probes and survival.

To determine whether the type of mutation is a prognostic factor independent of the DNA methylation subtypes, we stratified these subtypes by *IDH1/2* mutation (IDH1^R132H^ vs. non-R132H IDH1/2 mutated). Our data show that, even within the prognostic DNA methylation subtypes, patients harbouring non-R132H IDH1/2-mutated tumours had a significantly longer survival compared to those harbouring IDH1^R132H^-mutated tumours, regardless of the classifier used (Fig. 2d, supplementary Fig. 1, Online resource). The type of *IDH1/2* mutation was also an independent prognostic factor in a multivariable analysis that included all known factors associated with survival in this trial (treatment, age, corticosteroid use and sex, supplementary Table 1, online resource). It remained significant when DNA methylation subclass was included in this analysis (Table 1, Supplementary Table 2, online resource). These data demonstrate that the type of *IDH1/2* mutation is an independent factor associated with patient survival.

**Fig. 3:**
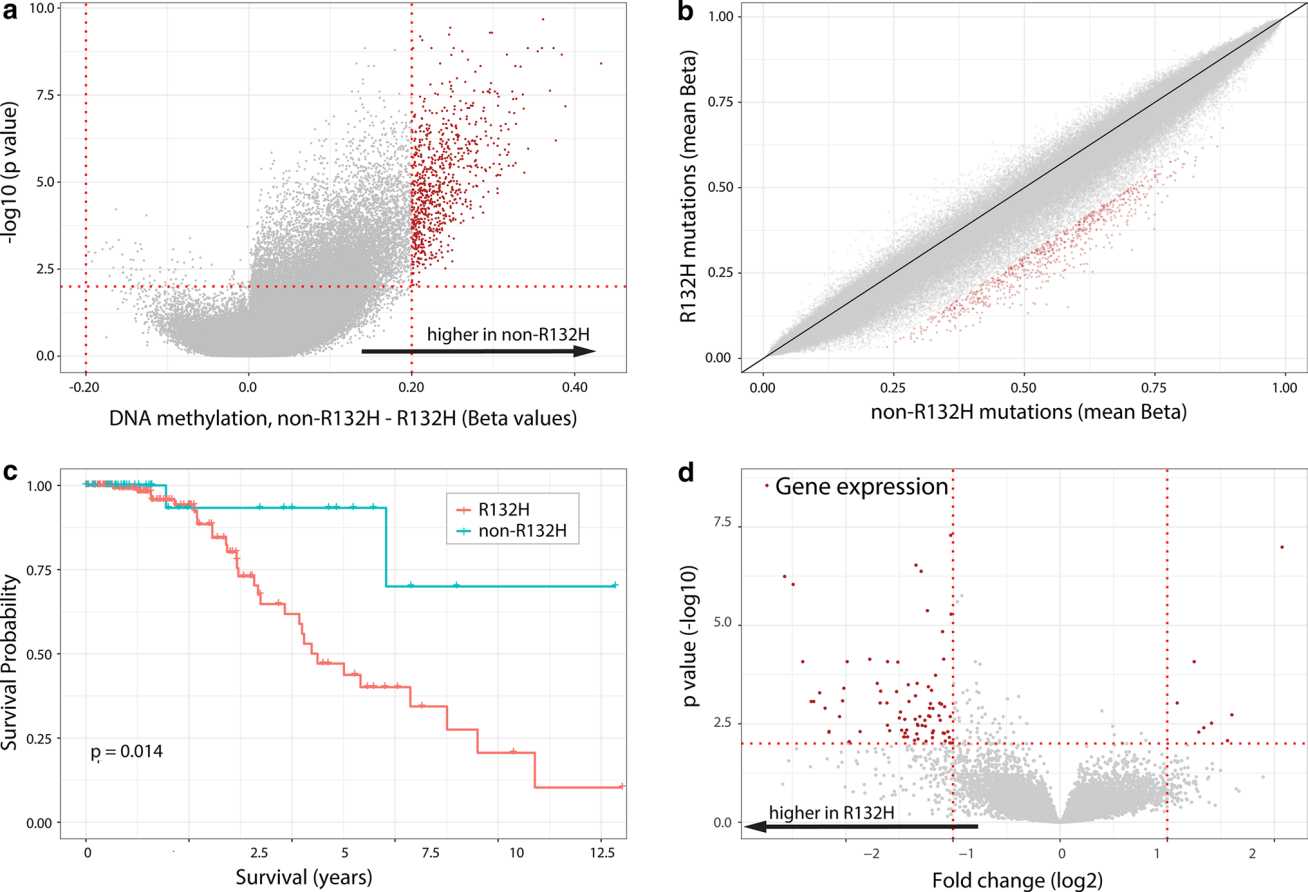
Non-R132H IDH1/2-mutations are associated with higher DNA methylation levels, lower gene expression and improved survival of 1p19q non-codeleted astrocytoma patients of the TCGA. Volcano plot (**a**) and XY plot (**b**) showing differences in methylation in non-R132H IDH1/2 vs. IDH1^R132H^ mutated tumours. **c** Patients harbouring non-R132H IDH1/2-mutated tumours have improved outcome. **d** Volcanoplot showing differential expression of genes between non-R132H IDH1/2 and IDH1^R132H^ mutated tumours. Most differentially expressed genes (red dots) have lower expression in non-R132H IDH1/2-mutated tumours (see also Supplementary Table 2, online resource)

To confirm these observations, we performed a similar analysis on the *IDH1/2* mutated, 1p/19q non-codeleted glioma patients included in the TCGA dataset [8]. Similar to observed in the CATNON dataset, a striking increase in DNA methylation levels was seen in non-R132H IDH1/2-mutated tumours compared to those harbouring a IDH1^R132H^ mutation (Fig. 3a, b). Also similar was the observation that patients harbouring non-R132H IDH1/2-mutated tumours survived significantly longer; the hazard ratio (HR) of patients harbouring non-R132H IDH1/2- mutated tumours (*n* = 37) versus IDH1^R132H^-mutated tumours (*n* = 177) was 0.20 (95% CI [0.047, 0.837], *p* = 0.028 Fig. 3c). Finally, IDH1^R132H^ mutated tumours also had a higher proportion of tumours assigned to the prognostically poorer G-CIMP low DNA methylation class (4/116 vs. 0/27) and a higher number at risk of progression to G-CIMP low (29/111 vs. 0/24, *p* = 0.016). The type of IDH mutation remained a factor significantly associated with survival in a multivariable model that contained tumour grade and patient age (supplementary Table 3, online resource).

DNA methylation generally shows a negative correlation with gene expression, especially when the methylated CpGs are located near the transcriptional start site [44, 50]. We, therefore, examined whether the reduction in DNA methylation in IDH1^R132H^ mutated tumours is associated with an increase in gene expression in the 1p/19q non-codeleted gliomas present in the TCGA dataset. Indeed, of the genes differentially expressed between IDH mutation types (with > twofold change in expression level at *p* < 0.01 significance level) in astrocytomas, most (157/183, 86%) were upregulated in IDH1^R132H^ mutated tumours (Fig. 3d, Supplementary Table 4, online resource). Pathway analysis using these 183 genes indicates that genes upregulated in IDH1^R132H^ mutated tumours were involved in cellular movement, cell death and survival, cell-to-cell signalling and interaction and carbohydrate metabolism (Supplementary Fig. 2, online resource).

**Fig. 4:**
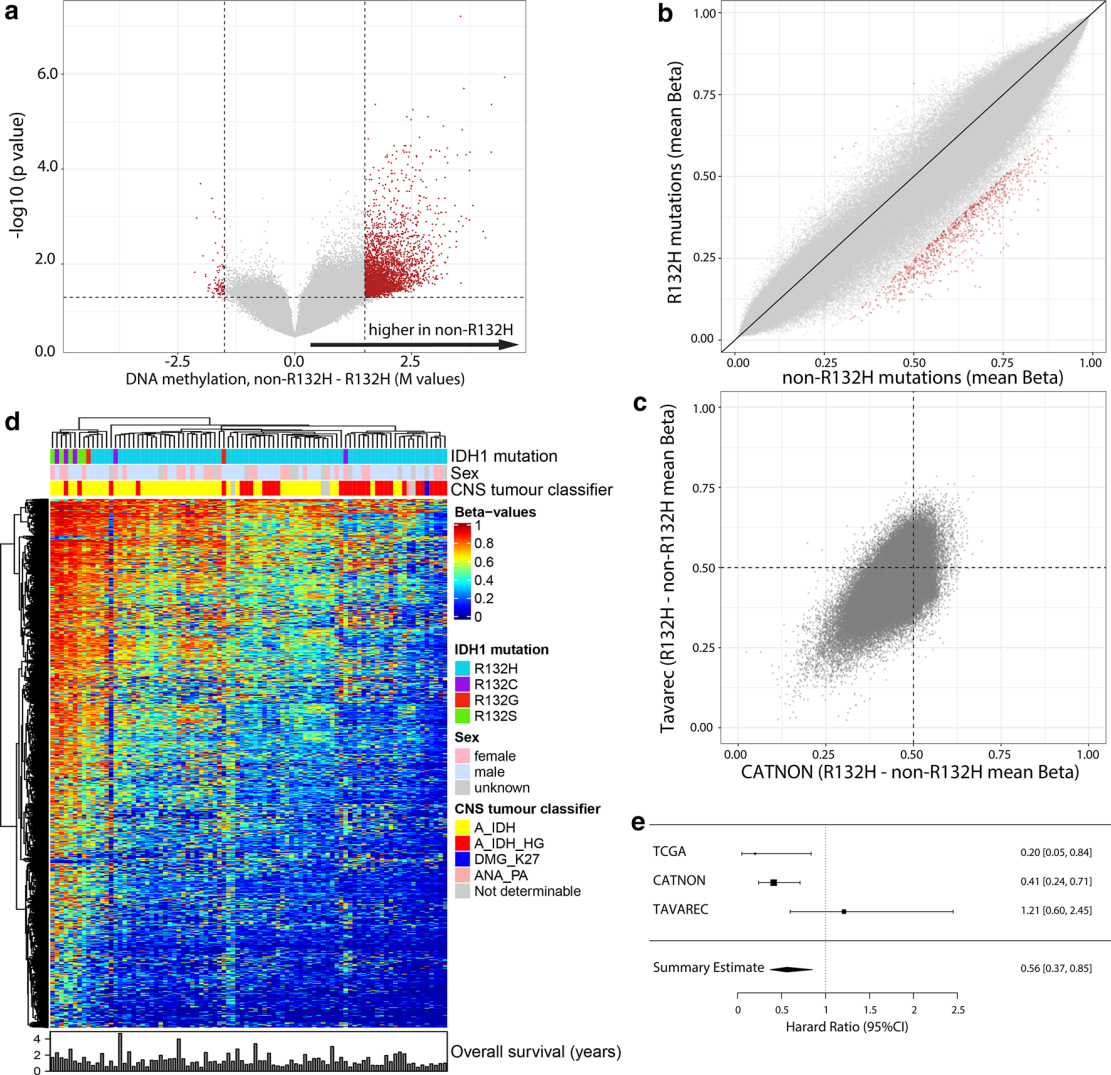
Non-R132H IDH1/2-mutations are associated with higher DNA methylation levels in 1p19q non-codeleted astrocytoma samples of patients included in the Tavarec trial. Volcano plot (**a**) and XY plot (**b**) showing differences in methylation in non-R132H IDH1/2 vs. IDH1^R132H^ mutated tumours. **c** Differential methylation between non-R132H IDH1/2 vs. IDH1^R132H^ mutated tumours showed a large degree of overlap in CATNON (x axis) and Tavarec (y axis) samples. **d** Heatmap of the most differentially methylated probes (red dots in **a** and **b**), shows a gradient in methylation levels. Non-R132H IDH1/2-mutated tumours cluster at the far left (high methylation), where poor prognostic methylation subtypes (epigenetics subtypes) cluster at the opposite end. **e** Forrest plot showing the summary HR estimate of 1p19q non-codeleted astrocytoma patients harbouring non-R132H IDH1/2 vs. IDH1^R132H^ mutated tumours

We performed a second validation using 1p/19q non-codeleted samples included in the randomised phase II TAVAREC clinical trial. Again, the vast majority of probes had lower DNA methylation levels in IDH1^R132H^ mutated tumours (*n* = 83) compared to non-R132H IDH1/2- mutated tumours (*n* = 11, Fig. 4a) and the most differentially methylated probes were those partially methylated in IDH1^R132H^ mutated tumours (Fig. 4b). Moreover, there was a large degree of overlap in differential DNA methylation between CATNON and TAVAREC samples (Fig. 4c). In TAVAREC, there was no significant difference in survival between patients harbouring IDH1^R132H^ and non-R132H IDH1/2-mutated tumours (HR 1.21, 95% CI [0.60, 2.45], *p* = 0.60). This, however, may be related to the specific inclusion criteria of this trial: patients were included only when the tumour showed signs of malignant progression at the time of progression (i.e. contrast enhancement on the MRI scan). In this respect, it is interesting to note that the percentage of non-R132H IDH1/2-mutated tumours was almost two-fold lower in TAVAREC trial samples (13%) compared to CATNON (19%) and TCGA (20%). Although this difference in frequency was not significant, these numbers are in line with the notion that non-R132H IDH1/2-mutated tumours have lower frequencies of malignant progression. The small number of patients harbouring non-R132H IDH1/2-mutated tumours (*n* = 11) may also mask potential survival differences. A heatmap of most differentially methylated probes shows that non-R132H IDH1/2-mutated tumours and tumours assigned to the prognostically poorer subclass A_IDH_HG clustered at opposite ends of this heatmap (Fig. 4d).

A forest plot of the combined CATNON, TCGA and TAVAREC survival data shows a summary estimate HR for non-R132H IDH1/2-mutated tumours of 0.56 with 95% CI [0.37, 0.85], association *p* = 0.006 (Fig. 4e).

**Fig. 5:**
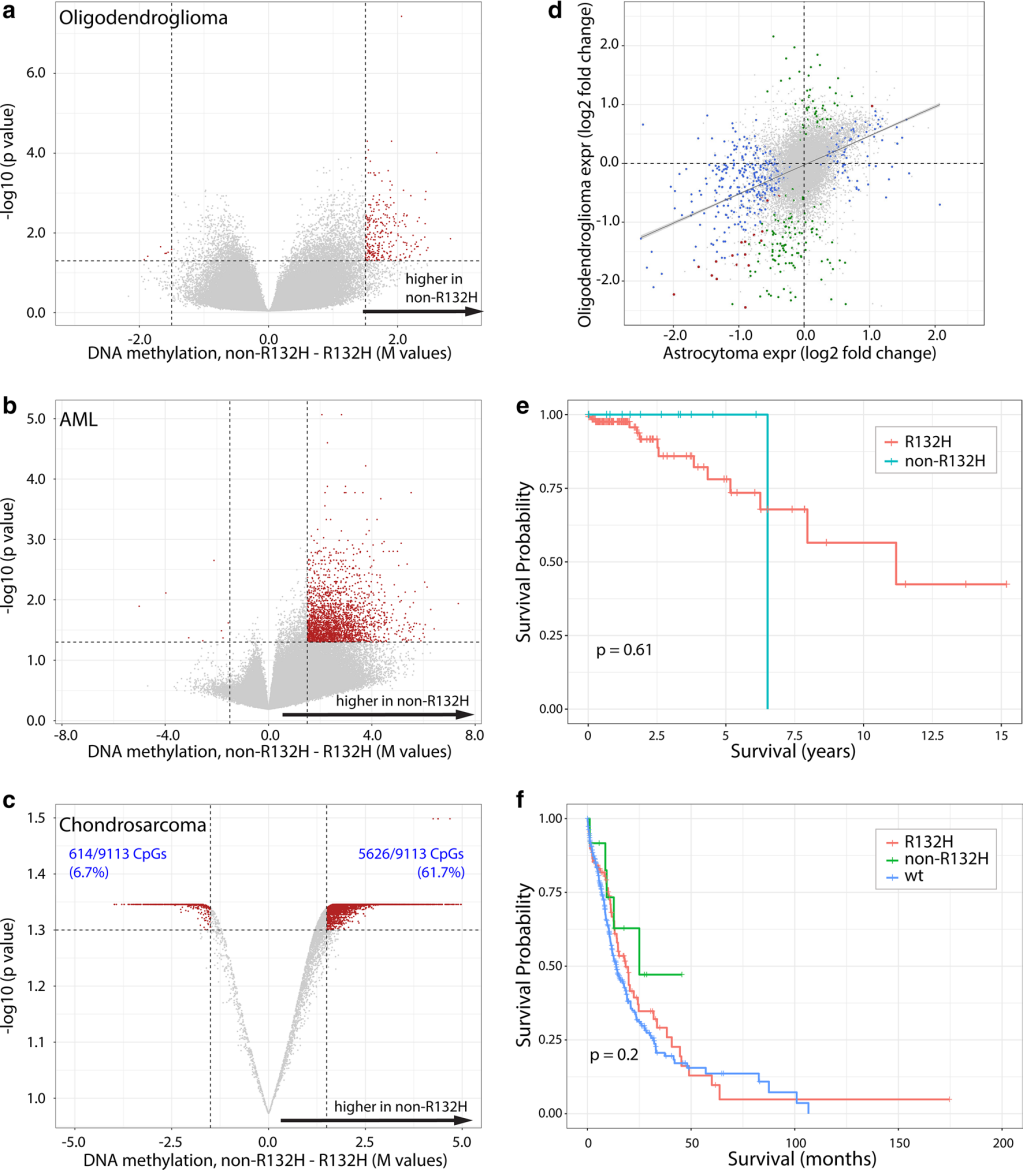
non-R132H IDH1/2-mutations are associated with higher DNA methylation levels independent of tumour type. Volcano plot of 1p19q codeleted oligodendrogliomas (**a**), AML (**b**) and chondrosarcomas (**c**) showing differences in methylation in non-R132H IDH1/2 vs. IDH1^R132H^ mutated tumours. Red dots depict CpGs that had a > 0.2 change in beta value and were significant (*p* < 0.01). Although the difference in chondrosarcomas is less than in other tumour types, the majority of significant CpGs was in non-R132H IDH1/2-mutated tumours (e.g. 225 CpG showed a > 0.3 increase in beta value at *p* < 0.01 where only 47 showed a similar decrease). **d** Gene expression differences between non-R132H IDH1/2 vs. IDH1^R132H^ mutated tumours in 1p19q non-codeleted astrocytomas (*x*-axis) and 1p19q codeleted oligodendrogliomas (*y*-axis) shows a large degree of overlap. Blue, green and red dots depict genes significantly differentially expressed in astrocytomas, oligodendrogliomas or both respectively (see also Supplementary Tables 2 and 3, online resource). **e** Survival of 1p19q codeleted oligodendroglioma patients present in the TCGA database harbouring non-R132H IDH1/2 vs. IDH1^R132H^ mutated tumours. There were too few events evaluate survival differences per mutation type. **f** Mutation type-specific survival differences in AML

To test whether mutation-dependent DNA methylation differences were restricted to 1p/19q non-codeleted gliomas (astrocytomas), we analysed the genome-wide methylation profiles of (i) *IDH1/2* mutated, 1p/19q codeleted gliomas (oligodendrogliomas, TCGA), (ii) acute myeloid leukemias (TCGA) and (iii) chondrosarcomas. Although the sample sizes of these datasets were relatively small in all tumour types (1p/19q codeleted gliomas *n* = 135 vs. 14; acute myeloid leukemias *n* = 4 vs. *n* = 24; chondrosarcomas *n* = 3 vs. *n* = 17 for IDH1^R132H^ and non-R132H IDH1/2-mutated tumours respectively), there was less DNA methylation in IDH1^R132H^ vs. non-R132H IDH1/2-mutation tumours (Fig. 5a–c). These data demonstrate that the level of DNA methylation is lower in tumours harbouring *IDH1/2* mutations with presumed low D-2HG production.

Gene expression analysis of 1p/19q codeleted gliomas present in the TCGA dataset identified 148 differentially expressed genes (expression fold change > 1 or < − 1 and *p* < 0.01). Similar to observed in astrocytic tumours, the majority of identified genes (123/148, 83%) were upregulated in IDH1^R132H^ mutated tumours (Supplementary Table 5, online resource). Moreover, there was a relatively large degree of concordance in differential expression between the two analyses (Fig. 5d) and sixteen genes were identified in both analyses.

The number of samples and events of the various datasets in patients with 1p/19q codeleted gliomas was insufficient to determine mutation type-dependent survival differences. For example, there were only 14 non-R132H IDH1/2-mutated 1p/19q codeleted tumours in the TCGA dataset, with only 1 event noted (in the IDH1^R132H^ mutated tumours there were 14 events in 135 patients). The HR for TCGA samples was 0.59 (95% CI [0.077, 4.595], *p* = 0.62, Fig. 5e). Also in the MSK-Impact [9] and the Chinese Glioma Genome Atlas (CGGA) [23] there were too few samples and events to determine survival benefit in patients harbouring non-R132H IDH1/2–mutated tumours. In these datasets, the events/number in non-R132H IDH1/2 vs. IDH1^R132H^ mutated samples was 0/6 vs. 3/34 and 0/5 vs. 3/31 in MSK impact, and CGGA datasets respectively. We were not able to determine survival differences in AML (*n* = 12 with 5 events vs. *n* = 89, 54 events, HR 1.49, 95% CI [0.59, 3.75], *p* = 0.39, Fig. 5f).

## Discussion

Our data shows that *IDH1/2mt* gliomas are distinct when compared to other *IDH1/2mt* tumours in that they have a disproportionally high percentage of IDH1^R132H^ mutations and raise the attractive clinical association between different rarer (codon 132) mutations and outcome. Patients harbouring IDH1^R132H^ mutated tumours have lower levels of genome-wide DNA methylation, regardless of tumour type (1p/19q non-codeleted gliomas, 1p/19q codeleted gliomas, AML and chondrosarcomas). For 1p/19q non-codeleted *IDH1/2mt* gliomas, this difference is clinically relevant as patients harbouring non-R132H IDH1/2-mutated tumours have improved outcome. Since IDH1^R132H^ mutations are presumed to be relatively poor in D-2HG production, our data are in line with the observation that glioma patients with higher D-2HG levels have improved outcome [34]. Our data are also in line with data from a meeting abstract showing similar mutation-specific survival differences [17].

The observation that patients harbouring non-R132H IDH1/2-mutated gliomas have longer survival is of importance for clinical practice as the specific *IDH1/2* mutation could alter patient prognostication. In this respect diagnostic assays should be able to discriminate between the type of IDH-mutation present; non-R132H IDH1/2-mutations comprise ~ 10% of all IDH-mutations in astrocytomas. Moreover, the efficacy of treatment with alkylating agents, IDH1/2 inhibitors, or other novel treatments might vary per mutation type, and therefore may be taken into account as a stratification factor in future clinical trials.

It has been reported that individual IDH1/2 mutations differ in their ability to produce D-2HG. In fact, the most common mutation in gliomas, IDH1^R132H^, is reported to be relatively inefficient in producing this oncometabolite [5, 40]. The differential capacity of IDH mutations in D-2HG production is supported by observations from cell lines and clinical samples where tumours harbouring the IDH1^R132H^ mutation generally have lower D-2HG levels compared to those with other IDH mutations [21, 24, 25, 29, 40] (but not in all [10]) though confounding factors such as tumour purity may influence these observations. Previous reports have shown that D-2HG is a weak inhibitor of TET2 enzymes as relatively high levels of D-2HG are required to inhibit the enzyme [31, 53]. In fact, the IC50 value for TET2 inhibition (~ 5 mM) is in the same range as the intratumoral D-2HG levels [10, 21, 29, 31]. As TET2 mediates the first step in DNA demethylation, lower D-2HG levels may result in reduced inhibition of DNA-demethylation. Therefore, although we did not directly measure D-2HG levels, the partial inhibition of TET2 may explain the lower overall methylation in IDH1^R132H^-mutated tumours.

The improved outcome of non-R132H IDH1/2-mutated astrocytomas may be explained by a reduced expression of genes that support tumour growth and/or induce treatment sensitivity caused by the increase in CpG methylation. Evidence supporting this hypothesis is the observation that many of the differentially expressed genes are involved in pathways associated with cancer. However, the improved outcome of non-R132H IDH1/2-mutated astrocytomas may also be related to the observation that D-2HG is toxic to cells, though only at high concentrations. For example, we have previously shown that exposure to D-2HG or expression of mutated IDH constructs reduced proliferation of cells, both in-vitro and in-vivo [6]. Later independent studies largely confirmed these observations and also conversely, reduction of D-2HG levels by mutant IDH inhibitors increased cell proliferation [18, 38, 40, 47, 55]. It should be noted, however, that in some preclinical model systems a growth inhibitory effect of IDH-inhibitors was observed [39, 41]. Functional experiments should confirm this hypothesis. Alternatively, differences in genetic stress and related mutational signatures may also explain the differential distribution of mutations in IDH [2, 3].

Apart from the type of IDH mutation present in the tumour, other prognostically relevant factors have also been described [43]. This includes histological tumour grade where patients with grade 2 astrocytomas have longer survival than those with grade 3 or grade 4 [36]. It should be noted, however, that we find that tumour grade is not a prognostic factor for the TCGA samples included in this study while the type of IDH-mutation is. In addition, the CATNON trial was performed on anaplastic (grade 3) tumours only.

Limitations of this study include the relatively small sample size of several datasets, especially those with a diagnosis other than the non-1p/19q codeleted gliomas. In addition, the absence of D-2HG level data limits the exploration of a direct correlation between *IDH1/2* mutation type and genome-wide DNA methylation.

In short, we described the effect of *IDH1/2* mutation type on patient outcome and the strong correlation between these specific mutations and genome-wide DNA methylation status. Our observation that non-R132H IDH1/2-mutated 1p/19q non-codeleted gliomas have a more favourable prognosis than their IDH1^R132H^ mutated counterpart is clinically relevant and should be taken into account for patient prognostication.

## Supplementary Information

Below is the link to the electronic supplementary material.

Supplementary file1 (TIF 2208 KB)

Supplementary file2 (TIF 11390 KB)

Supplementary file3 (DOCX 36 KB)
