# Supplementary figures and images for: Non-IDH1-R132H IDH1/2 mutations are associated with increased DNA methylation and improved survival in astrocytomas, compared to IDH1-R132H mutations
Source: Acta Neuropathol. 2021 Mar 19;141(6):945–57. doi: 10.1007/s00401-021-02291-6 (PMC8113211; doi:10.1007/s00401-021-02291-6)

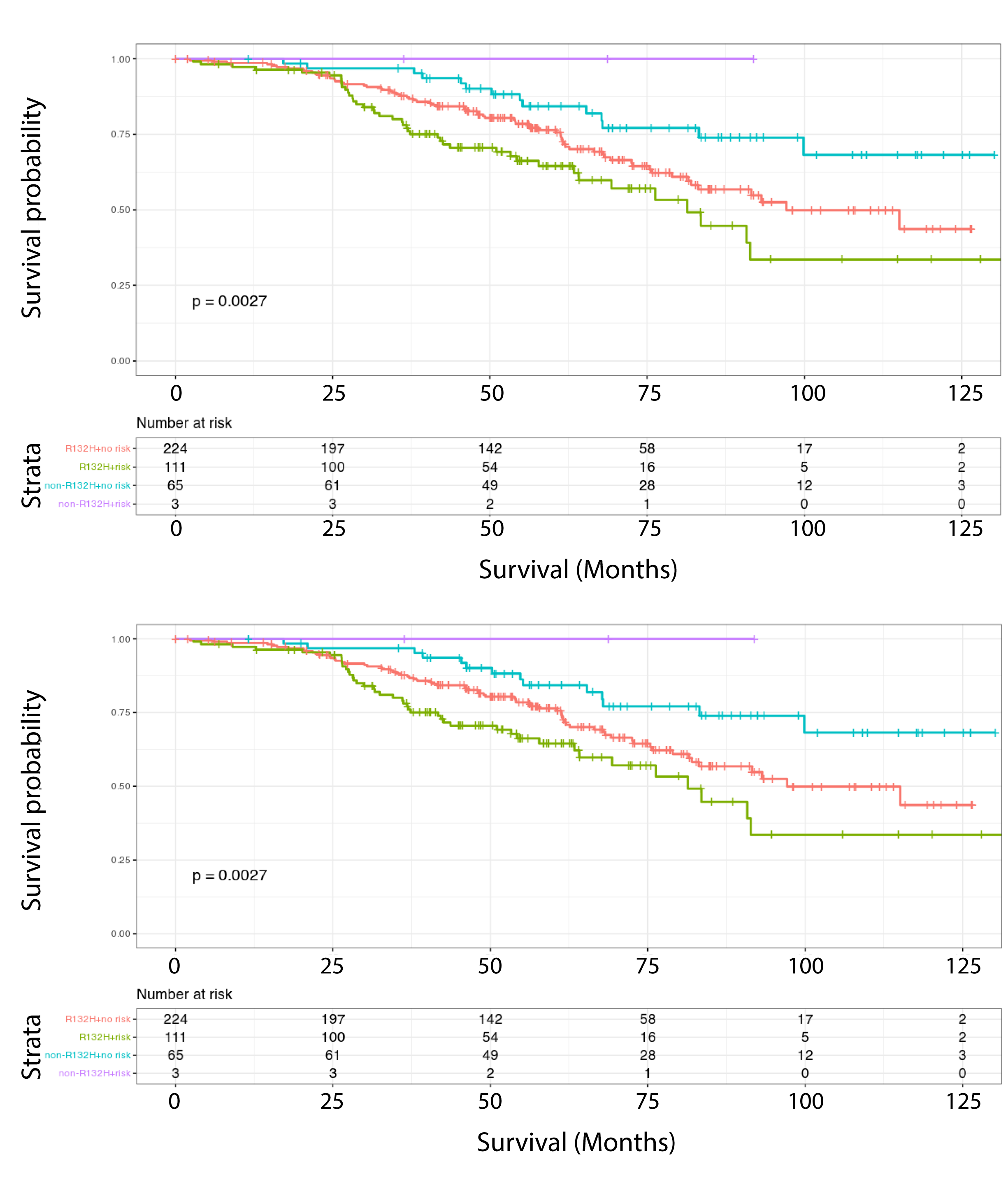

Supplement: Supplementary file 1 — Supplementary file1 (TIF 2208 KB) [file 401_2021_2291_MOESM1_ESM.tif]

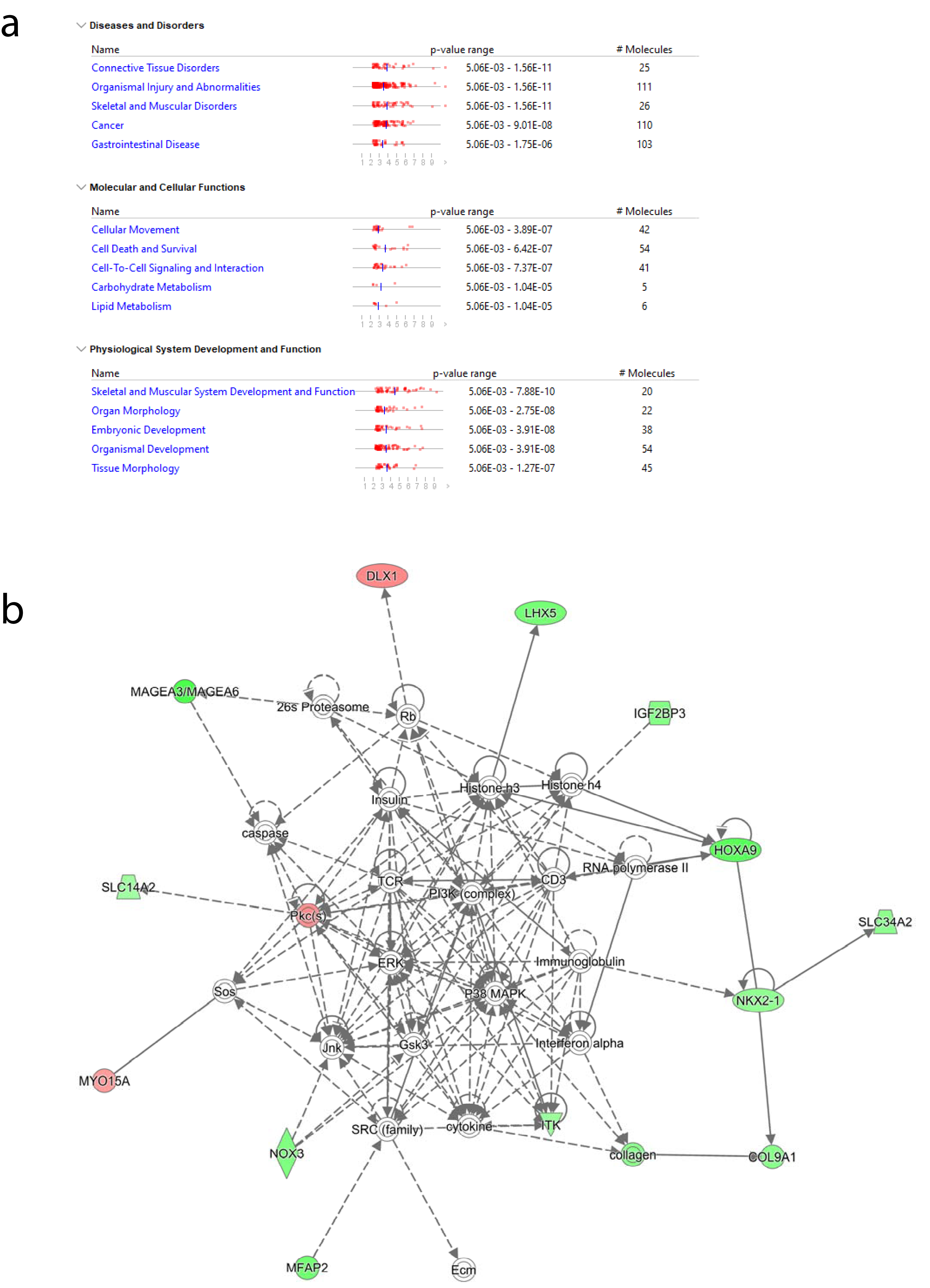

Supplement: Supplementary file 2 — Supplementary file2 (TIF 11390 KB) [file 401_2021_2291_MOESM2_ESM.tif]
